# Supplementary material for: Complexome profiling of the Chlamydomonas psb28 mutant reveals TEF5 as an early PSII assembly factor
Source: Plant Cell. 2025 Mar 17;37(6):koaf055. doi: 10.1093/plcell/koaf055 (PMC12164590; doi:10.1093/plcell/koaf055)
Supplement: koaf055_Supplementary_Data [file koaf055_supplementary_data.zip › psb28_interactive.zip!/plots/intensity/qualityPlots/peptides/CPLD42_Cre01.g004450.t1.2.html]

### Cre01.g004450.t1.2

Trivial name: CPLD42  
SeqCoverage [median]: 11.90  
Mass: 29.81 kDa  
  
 

| Sequence | MissedCleavage | Mass [Da] | wtIntensity | muIntensity | Score |
| --- | --- | --- | --- | --- | --- |
| FKEETFSNVDGR | 1 | 1427.67 | 389.09 | 0.00 | 35.20 |
| VNSAADLKK | 1 | 944.53 | 0.00 | 346.26 | 115.71 |
| VQVGMFGDVK | 0 | 1078.55 | 0.00 | 559.09 | 67.90 |
| VQVGMFGDVKK | 1 | 1206.64 | 0.00 | 530.56 | 46.59 |
|  |  |  | 389.1 | 1435.9 |  |
